# Ars2‐containing bispecific, Fab‐ and IgG1‐format BAR‐bodies to target DLBCL cells

**DOI:** 10.1002/jha2.635

**Published:** 2022-12-27

**Authors:** Maximilian Kiefer, Lorenz Thurner, Theresa Bock, Onur Cetin, Igor Kos, Vadim Lesan, Dominic Kaddu‐Mulindwa, Joerg Thomas Bittenbring, Natalie Fadle, Evi Regitz, Markus Hoth, Frank Neumann, Klaus‐Dieter Preuss, Michael Pfreundschuh, Konstantinos Christofyllakis, Moritz Bewarder

**Affiliations:** ^1^ Internal Medicine I Saarland University Medical Center Homburg Germany; ^2^ Biophysics, CIPMM Saarland University Homburg Germany

**Keywords:** autoantigen, BAR‐bodies, B cells, B‐cell receptor, lymphoid malignancies

## Abstract

Despite recent advances in the therapy of diffuse large B‐cell lymphoma, not otherwise specified (DLBCL), around 30% of patients develop refractory disease or relapse after first‐line treatment. Recently, Ars2 was reported as the auto‐antigenic target of the B‐cell receptor (BCR) in approximately 25% of activated B‐cell DLBCL cases. Ars2 could be used to specifically target B cells expressing Ars2‐reactive BCRs. However, the optimal therapeutic format to integrate Ars2 into has yet to be determined. To mimic therapeutic antibody formats, Ars2‐containing bispecific and IgG1‐like constructs (BCR antigens for reverse [BAR]‐bodies) were developed. Two bispecific BAR‐bodies connecting single‐chain antibodies against CD16 or CD3 to the BCR‐binding epitope of Ars2 were constructed. Both constructs showed strong binding to U2932 cells and induced effector cell‐dependent and selective cytotoxicity against U2932 cells of up to 44% at concentrations of 20 μg/ml. Additionally, IgG1‐format Ars2 BAR‐bodies were constructed by replacing the variable heavy‐ and light‐chain regions of a full‐length antibody with the Ars2 epitope. IgG1‐format Ars2 BAR‐bodies also bound selectively to U2932 and OCI‐Ly3 cells and induced selective cytotoxicity of up to 60% at 10 μg/ml. In conclusion, Ars2‐containing bispecific and IgG1‐format BAR‐bodies both are new therapeutic formats to target DLBCL cells.

## 1 INTRODUCTION

Not otherwise specified diffuse large B‐cell lymphoma (DLBCL) is the most common lymphatic neoplasia and accounts for 25%–35% of adult non‐Hodgkin lymphomas [1, 2, 3]. After first‐line therapy, up to 15% of patients show refractory disease with an additional 20%–25% experiencing relapse within the first 2 years [4]. In patients with refractory DLBCL, survival outcomes are particularly poor, and further treatment options are needed. Gene expression profiling has identified three distinct DLBCL subtypes according to the assumed cell of origin: activated B‐cell like (ABC), germinal center B‐cell like, and unclassified. Dysregulated B‐cell receptor (BCR) signaling seems to play a key role, especially, in the ABC subtype with constant NF‐κB pathway activity. In some ABC DLBCLs, this NF‐κB activity results from CARD11 mutations (10% of ABC DLBCLs) but in most ABC DLBCLs NF‐κB activity stems from “chronic active” BCR signaling that is assumed to be triggered by antigen contact of the BCR [5]. Recent studies further refined the molecular classification of DLBCLs into at least six genetic subtypes, and it can be speculated that some of them may be driven by the antigenic stimulus of the BCR [6, 7, 8]. Antigenic BCR stimulation by autoantigens has been proposed as pivotal pathway for malignancy in several types of lymphoma. We have identified and characterized several autoantigens as the specific targets of BCRs or the paraprotein of different B‐cell malignancies [9, 10, 11, 12, 13, 14, 15, 16]. Ars2 was reported as the autoantigenic target of the BCR in approximately 25% of all ABC‐type DLBCL cases [17]

The BCR of lymphoma cells represents an ideal target for new therapeutic approaches and different BCR‐targeting therapeutic formats like anti‐idiotype antibodies and peptibodies have been developed with moderate clinical success [18, 19, 20, 21, 22, 23]. Similarly, our group reported of an approach to target the BCR of a malignant B‐cell clone via identified common BCR antigens that was designated as BAR (BCR antigens for reverse targeting). Constructs integrating BARs into antibody‐like formats have been termed BAR‐bodies [16]

In this work, we describe the integration of Ars2 into three different therapeutic formats: bispecific BAR‐bodies, Fab‐format BAR‐bodies, and IgG1‐format BAR‐bodies.

## 2 MATERIALS AND METHODS

The cloning strategies for all constructs are described in the supplement and used primers are shown in tabels 1, 2, 3.

**TABLE 1:** Primers used for cloning of bispecific Ars2 BAR‐bodies

	Primer name	Sequence (5′–3′)
αCD3 single‐chain fragment (scFv), heavy chain	αCD3‐VH‐HindIII (sense) αCD3‐VH‐BamHI (antisense)	AAGCTTGCCACCATGCAGGTCCAGCTGCAGCAG GGATCCACCACCACCGGAGCCGCCGCCGCCAGAACCACCACCACCAGAACCACCACCACCTGTTGTTTTGGCTGAGGA
αCD3 single‐chain fragment (scFv), light chain	αCD3‐VK‐BamHI (sense) αCD3‐VK‐EcoRI (antisense)	GGATCCCAAATTGTTCTCACCCAGTC GAATTCGATCCGCCACCGCCAGAGCCACCTCCGCCTGAACCGCCTCCACCAGTTGGTGCAGTATCAGCC
ARS2 epitope	ARS2‐EcoRV (sense) ARS2‐SmaI‐His (antisense)	GATATCATGAAGGAAGCCAAAAAGAGTAGC CCCGGGTTAATGGTGGTGGTGATGATGAGCGCTCTCTGACTCCGACTCAGAC

**TABLE 2:** Primers used for cloning of Fab‐format BAR‐bodies

	Primer name	Sequence (5′–3′)
Version A, light chain	Ars2 Fab AA343 ApaL1—sense	AGT GCA CAG AAG GAA GCC AAA AAG AGT AGC
	Ars2 Fab AA466 Xho1—antisense	CTC GAG CCA GCC ACG ACG GAA AAA C
Version A, heavy chain	Ars2 Fab AA343 Nco1—sense	CCA TGG CCC AAG GAA GCC AAA AAG AGT AGC
	Ars2 Fab AA466 BstE2—antisense	GGT CAC CCA GCC ACG ACG GAA AAA C
Version B, light chain	Ars2 Fab AA290 ApaL1—sense	AGT GCA CAG GCT GGA GCA GGC CTA GGG
	Ars2 Fab AA410 Xho1—antisense	CTC GAG CAG CCC CGC GGC GTC CTT G
Version B, heavy chain	Ars2 Fab AA290 Nco1—sense	CCA TGG CCG CTG GAG CAG GCC TAG GG
	Ars2 Fab AA410 BstE2—antisense	GGT CAC CAG CCC CGC GGC GTC CTT G
Version C, light chain	Ars2 Fab AA260 ApaL1—sense	AGT GCA CAG ACG GAG AAT GAT CTT CGT
	Ars2 Fab AA375 Xho1—antisense	CTC GAG CTC TGA CTC CGA CTC AGA C
Version C, heavy chain	Ars2 Fab AA260 Nco1—sense	CCA TGG CCA CGG AGA ATG ATC TTC GT
	Ars2 Fab AA375 BstE2—antisense	GGT CAC CTC TGA CTC CGA CTC AGA C

**TABLE 3:** Primers used for cloning of IgG1‐format BAR‐bodies

	Primer name	Sequence (5′–3′)
Light‐chain sense	Ars2 IgG1 AA343 Age1—sense	ACC GGT AAG GAA GCC AAA AAG AGT AGC
Light‐chain antisense	Ars2 IgG1 AA466 Nru1—antisense	TCG CGA TCC AGC CAC GAC GGA AAA AC
Heavy‐chain sense	Ars2 IgG1 AA343 Mun1—sense	CAA TTG AAG GAA GCC AAA AAG AGT AGC
Heavy‐chain antisense	Ars2 IgG1 AA466 Apa1—antisense	GGG CCC GCC GCC GCC GTT TAC CCG GAG ACA GGG A

### 2.1 Bacteria, cell lines, and cell culture

The *Escherichia coli* strain DH5α was used for general cloning steps and was obtained from Thermo Scientific (Waltham, MA, USA). For expression and purification of the different BAR‐body constructs, the *E. coli* strain TG1 with the chromosomal genotype SupE hsd ∆5 thi ∆5lac‐proAB) was used (Deutsche Sammlung von Mikroorganismen und Zellkulturen, DSMZ, Braunschweig, Germany).

For eukaryotic protein expression, HEK‐293 cells were used (DSMZ, Braunschweig, Germany). The DLBCL cell line HBL‐1 was kindly provided by the Georg August University of Göttingen. The U2932 cell line was kindly provided by M‐L Hansmann (Senckenberg Institute of Pathology, Frankfurt, Germany). OCI‐Ly3 cells and the mantle cell lymphoma (MCL) cell lines MAVER‐1 and Granta‐519 were purchased from DSMZ (Braunschweig, Germany). For authentication, the concordance of the VH gene sequences with published sequences was demonstrated by PCR analysis and sequencing. Cells were cultured in RPMI 1640 Medium (PAN‐Biotech, Aidenbach, Germany) with 4 mmol/l glutamine and 10% FCS. Peripheral blood mononuclear cells (PBMCs) were prepared from control blood samples after centrifugation with Ficoll (1500 rpm for 3 min) and used 2 days after preparation.

### 2.2 Flow cytometry

Binding of bispecific, Fab‐format, and IgG1‐format Ars2 BAR‐bodies to lymphoma cell lines was assessed by flow cytometry. For all flow cytometric analyses, a BD FACS Canto Flow Cytometer (BD Biosciences, Heidelberg, Germany) was used. For data analysis and visualization, the Kaluza software (Beckman Coulter GmbH, Krefeld, Germany) was used.

Bispecific constructs were assessed on the MCL cell line MAVER‐1 and the DLBCL cell line U2932. Approximately 5 × 10^6^ lymphoma cells (1 × 10 [6]/ml) were incubated for 45 min at RT with the recombinant proteins at a concentration of 1 μg/ml, followed by washing steps. Thereafter, lymphoma cells were dyed with PE‐labeled anti‐His antibody for 30 min at RT.

Binding of Fab‐format Ars2 BAR‐bodies to Ars2‐reactive BCRs was assessed on U2932 and HBL‐1 DLBCL cell lines. A number of 1 × 10^5^ cells were incubated with 10 μg construct for 20 min at RT. Staining was done using the Penta‐His Antibody mouse monoclonal IgG1 (Qiagen, Hilden, Germany), 1:500, as primary antibody and a biotinylated anti‐mouse antibody (Dianova, Hamburg, Germany), 1:200, with streptavidin‐PE (Dianova, Hamburg, Germany), 1:500, as secondary system.

Binding of IgG1‐format Ars2 BAR‐bodies to Ars2‐reactive BCRs was assessed on U2932, OCI‐Ly3, Granta‐519, MAVER‐1, and HBL‐1 cell lines. Approximately 1 × 10^5^ lymphoma cells were incubated with 10 μg construct for 20 min at RT. Cells were then incubated with ANTI‐FLAG M2 antibody (Merck, Karlsruhe, Germany), 1:500, for 20 min at RT. Goat F(ab′)2 anti‐Mouse IgG (H+L)‐FITC (DIANOVA GmbH, Landwehr 2, 22087 Hamburg) 1:500 was used for secondary staining.

### 2.3 Cytotoxicity assay

Ars2 BAR‐body mediated cell lysis (with and without effector cells) was detected by LDH release assays. Cytotoxicity Detection Kit^PLUS^ (LDH) by Roche Applied Science (Penzberg, Germany) was used for all cytotoxicity analyses. Before analysis, all cells were washed 2× with RPMI 1640 medium (centrifugation at 1500 rpm for 3 min). All experiments were performed in triplicate. Each well contained 100 μl medium and approximately 5000 target cells (OCI‐Ly3, U2932, HBL‐1, MAVER‐1, Granta‐519).

The bispecific BAR‐bodies were evaluated for efficacy on U2932 and MAVER‐1 cells at different concentrations (20, 10, 1, and 0.1 μg/ml) after addition of effector cells (PBMCs) at an effector to target cell ratio (E:T) of 10:1 (50,000 PBMCs per well).

For the analysis of the IgG1‐format Ars2 BAR‐body, the OCI‐Ly3, U2932, HBL‐1, MAVER‐1, and Granta‐519 cell lines were used. For U2932 and HBL‐1 cells, the constructs were assessed on target cells without effector cells at concentrations of 10, 1.0, 0.5, and 0.1 μg/ml. Then, the constructs were evaluated after the addition of effector cells (PBMCs) at an E:T ratio of 10:1, corresponding to 50,000 effector cells. Ars2 and LRPAP1 BAR‐body in the IgG format were used on OCI‐Ly3, MAVER‐1, and Granta‐519 cells only in the presence of effector cells. Maximal and minimal lyses were used as controls. For determination of maximal lysis, 0.5% Triton X‐100 (Merck, Darmstadt, Germany) was added to target cells. For minimal lysis, no effector cells and no BAR‐bodies were added.

Plates incubated for 24 h at 37°C and 5% CO_2_. Next, the reaction reagent was added and incubated for 10 min. The reaction was stopped by hydrochloric acid. The final results were analyzed with the use of a Wallac Victor2 1420 Multilabel Counter by Applied Biosystems (CA, USA). The measurements were done at 490 nm. Specific lysis (%) was defined as (experimental lysis minus minimal lysis)/(maximum lysis minus minimal lysis) × 100. Visualization was performed with Prism 9, Version 9.4.1 (GraphPad Software, LLC).

## 3 RESULTS

### 3.1 Expression and binding properties of bispecific Ars2 BAR‐bodies

Bispecific Ars2 BAR‐bodies recruiting either CD3‐positive T‐cells or CD16‐positive NK cells were detected by western blot analysis with approximately 55 kD (Figure S1). They evaluated for binding to U2932 DLBCL cells known to express an Ars2‐reactive BCR. As negative control, the MCL cell line MAVER‐1 was used. Additionally, LRPAP1‐ETA, a toxin‐linked MCL‐specific BCR‐antigen, was investigated for binding properties against both cell lines.

**FIGURE 1:**
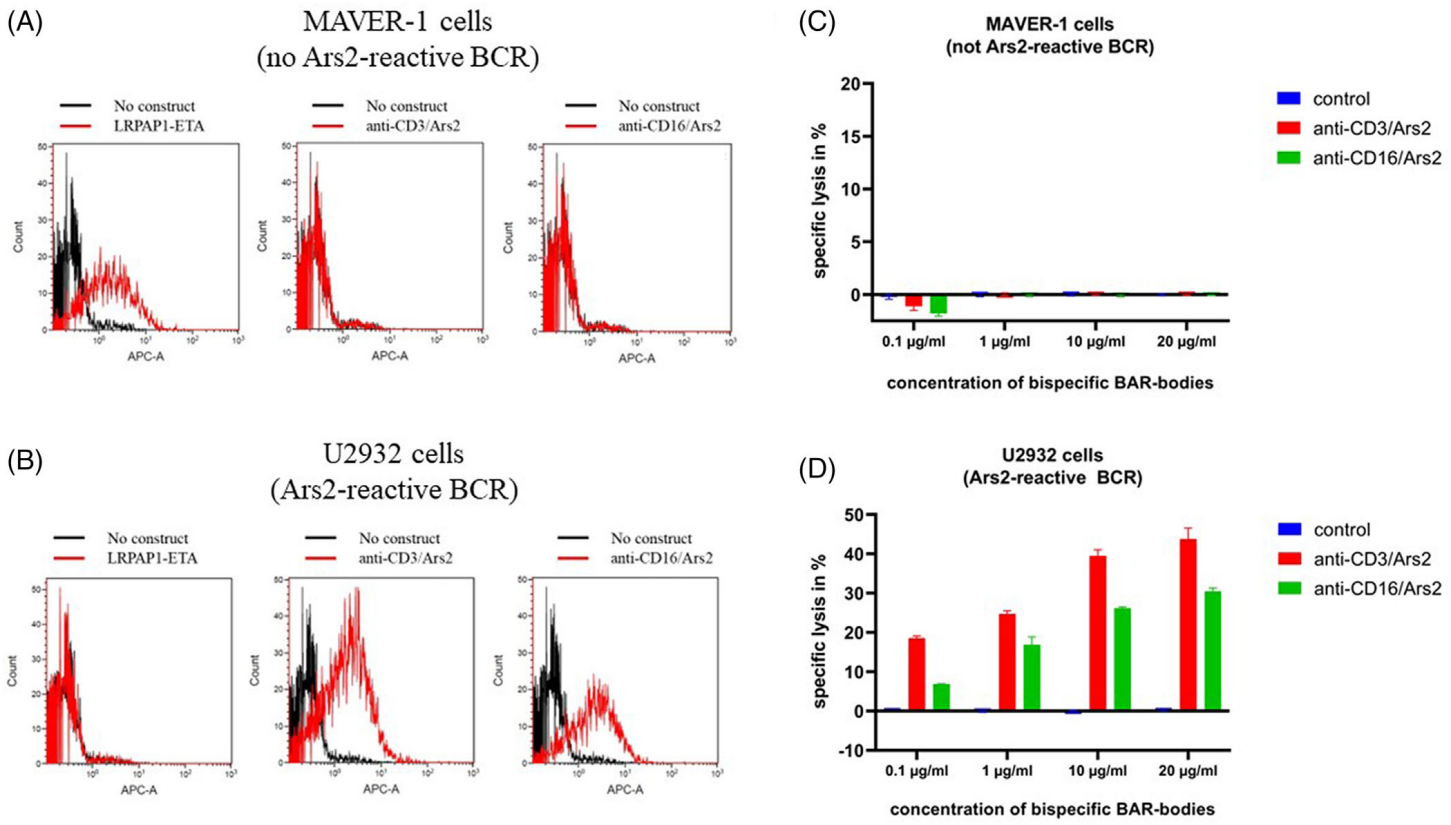
Characterization of bispecific Ars2 BAR‐bodies. Flow cytometric assessment of both bispecific Ars2 BAR‐bodies are shown in parts (A) and (B). Anti‐CD16/Ars2 and anti‐CD3/Ars2 both show no binding to cells of the mantle cell lymphoma (MCL) cell line MAVER‐1 expressing LRPAP1‐reactive B‐cell receptors (BCRs) (A). The LRPAP1/ETA′ immunotoxin served as positive control. Anti‐CD16/Ars2 and anti‐CD3/Ars2 show strong binding to U2932 cells expressing Ars2‐reactive BCRs, whereas LRPAP1/ETA′ does not bind to U2932 cells (B). When incubated with peripheral blood mononuclear cells (PBMCs) as effector cells, both bispecific BAR‐bodies had no effect on MAVER‐1 cells at any concentration (C) but induced lysis of U2932 cells in a concentration‐dependent manner (D). All experiments were performed in triplicate (error bars).

Both bispecific Ars2 BAR‐bodies demonstrated strong binding capacity to U2932 cells but not to cells of the MCL cell line MAVER‐1. LRPAP1‐ETA bound to MAVER‐1 cells expressing an LRPAP1‐reactive BCR [29]. but not to U2932 cells (Figure 1A,B).

### 3.2 Effector functions of bispecific Ars2 BAR‐bodies

In a next step, the bispecific constructs were investigated for their ability to induce PBMC‐mediated selective cytotoxicity in lymphoma cells. Both the CD3 and CD16 recruiting constructs were incubated for 24 h with MAVER‐1 and U2932 cells at increasing concentrations from 0.1 to 20 μg/ml. PBMCs were added at an effector to target cell ratio (E:T) of 10:1. Both bispecific Ars2 BAR‐bodies had no effect on MAVER‐1 cells (Figure 1C) but elicited specific cytotoxicity in U2932 cells in a concentration‐dependent manner when incubated with PBMCs (Figure 1D). Anti‐CD16/Ars2‐induced specific lysis ranged from 7% to 30% at concentrations of 0.1 and 20 μg/ml. Anti‐CD3/Ars2 conferred similar specific lysis on U2932 cells ranging from 19% to 44% at concentrations of 0.1 and 20 μg/ml, respectively.

### 3.3 Expression of Fab‐format Ars2 BAR‐bodies

**FIGURE 2:**
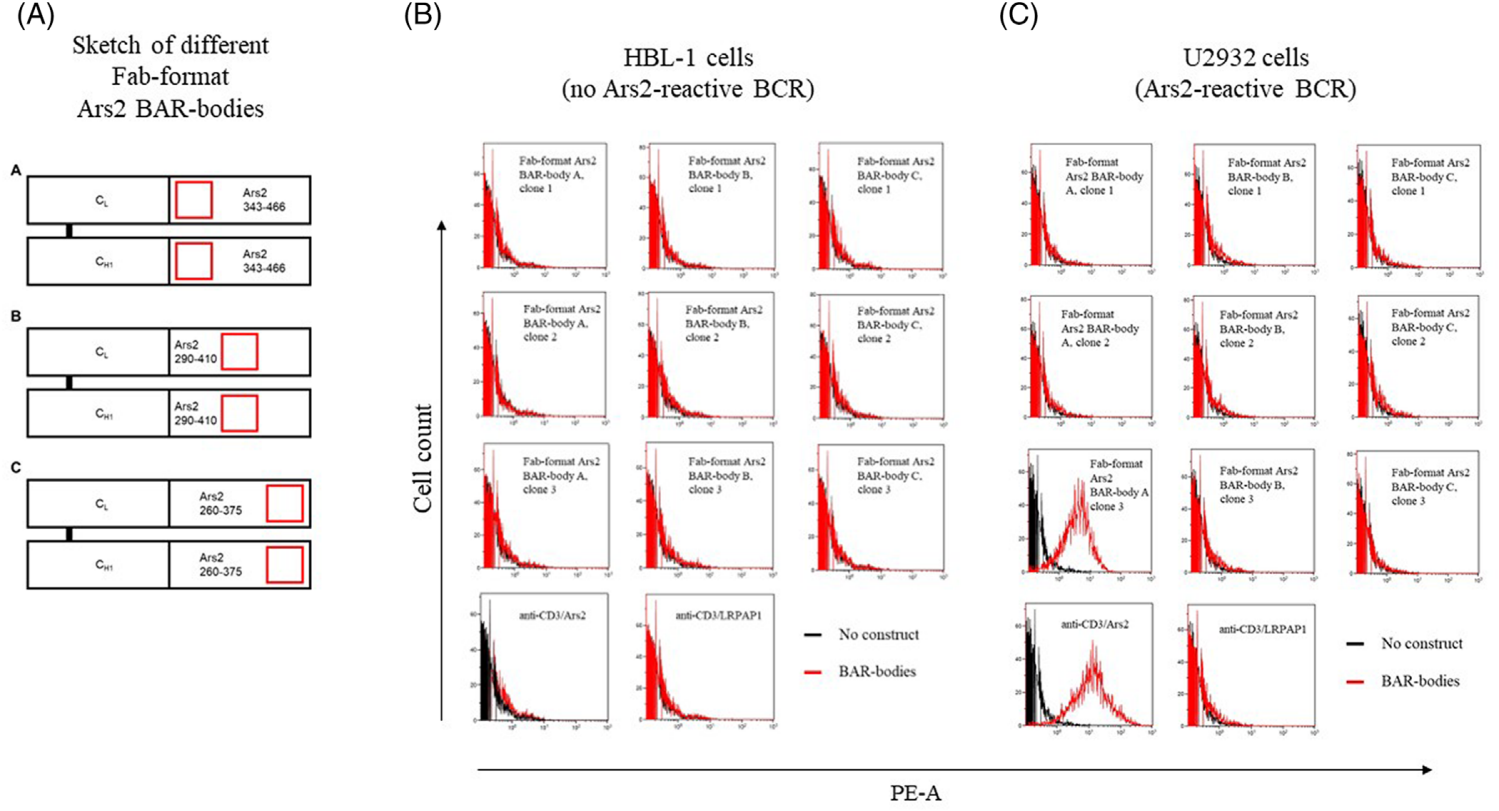
Sketch of three different versions of Fab‐format Ars2 BAR‐bodies (A). The constant region of the light chain (CL) and constant region 1 of the heavy chain (CH1) are connected with three Ars2 BAR regions that differ in their orientation of the B‐cell receptor (BCR)‐binding epitope of Ars2 spanning the aa 343–375 (depicted as red squares). Fab‐format Ars2 BAR‐body A includes the Ars2 amino acids 343–466, Fab‐format Ars2 BAR‐body B incorporates the Ars2 amino acids 290–410, and the BAR region of Fab‐format Ars2 BAR‐body C spans from the Ars2 amino acids 260–375. Fab‐format Ars2 BAR‐bodies were evaluated for binding to HBL‐1 cells, which express a BCR of unknown reactivity. No binding was observed (B). Fab‐format Ars2 BAR‐bodies were evaluated for binding to U2932 cells, which express a BCR with reactivity against the BAR Ars2. Clone 3 of Fab‐format Ars2 BAR‐body, version A, showed strong binding to U2932 cells (C). We used the bispecific anti‐CD3/Ars2 BAR‐body as positive control and the bispecific anti‐CD3/LRPAP1 BAR‐body as negative control.

Next, we aimed to construct Fab‐format BAR‐bodies in which the variable regions of the heavy and light chains are replaced with the BCR‐binding epitope of Ars2 (aa 343–375) to form a BAR region. Because the Ars2 epitope comprises 33 amino acids and regular immunoglobulin variable regions are approximately 120 amino acids long, the Ars2 epitope was elongated either on the 5′ end, the 3′ end, or both ends to match the length of regular variable regions. Three different Fab‐format BAR‐bodies were constructed which all included the BCR‐binding epitope of Ars2 (Figure 2A):

Fab‐format BAR‐body A (Ars2 aa 343–466), Fab‐format BAR‐body B (Ars2 aa 290–410), and Fab‐format BAR‐body C (Ars2 aa 260–375). All three versions of the Fab‐format Ars2 BAR‐body were expressed in TG1 *E. coli* bacteria and could be detected via western blot analysis with a molecular weight of 40 kDa (Figure S2).

### 3.4 Binding capacity of Fab‐format Ars2 BAR‐bodies

**FIGURE 3:**
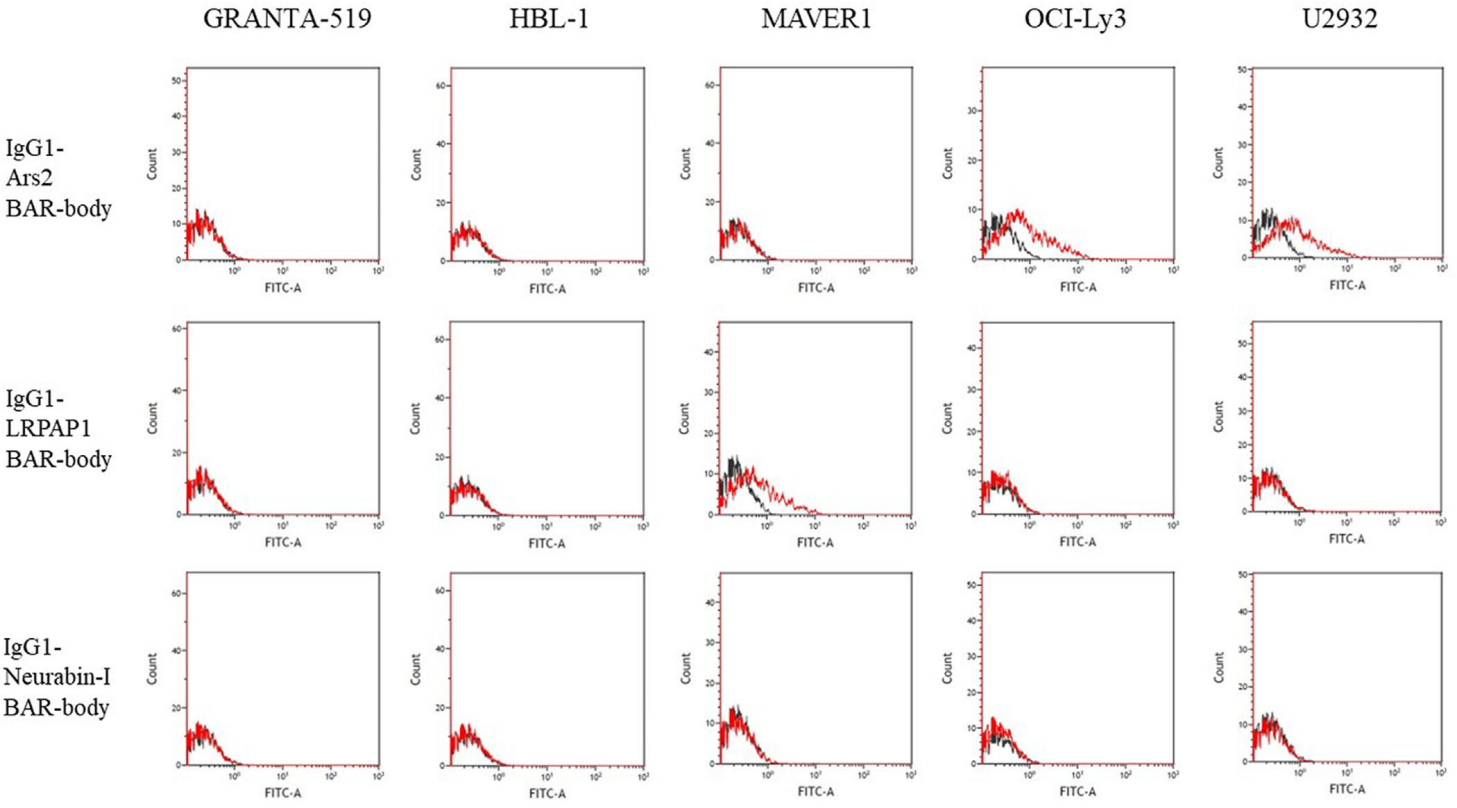
IgG‐format BAR bodies incorporating Ars2, LRPAP1, and Neurabin‐I were tested for binding to the diffuse large B‐cell lymphoma (DLBCL) cell lines U2932, OCI‐Ly3 (Ars2‐reactive B‐cell receptor [BCR]), and HBL‐1 (no Ars2‐reactive BCR) as well as to the mantle cell lymphoma (MCL) cell lines Granta‐519 (BCR of unknown reactivity) and MAVER‐1 (LRPAP1‐reactive BCR). Ars2 BAR‐bodies showed binding to OCI‐Ly3 and U2932 cells but not to other cell lines. LRPAP1 BAR‐bodies served as positive control and showed binding to MAVER‐1 cells as they express BCRs with known LRPAP1 reactivity. Neurabin‐I BAR‐bodies were used as negative controls and showed no binding to any used cell line.

All nine clones (three clones for each of the Fab‐format BAR‐bodies A, B, and C) of the recombinantly expressed Fab‐format Ars2 BAR‐bodies were tested for their binding capacity to the DLBCL cell lines U2932 (BCR with Ars2‐reactivity) and HBL‐1 (BCR with no Ars2‐reactivity) by flow cytometry (Figure 3). The bispecific BAR‐bodies anti‐CD3/LRPAP1 [24]. and anti‐CD3/Ars2 were used as negative and positive control, respectively. No binding of any Fab‐format BAR‐body or of the bispecific constructs to HBL‐1 cells was observed (Figure 2B). Clone 3 of the Fab‐format Ars2 BAR‐body A showed specific binding to U2932 cells, whereas clones 1 and 2 of the Fab‐format Ars2 BAR‐body A did not (Figure 2C). The BAR region of Fab‐format Ars2 BAR‐body A spans the amino acids 343–466 of the Ars2 protein. The bispecific anti‐CD3/Ars2 BAR‐body but not the anti‐CD3/LRPAP1 BAR‐body showed binding to U2932 cells (Figure 2C). Clone 3 of the Fab‐format Ars2 BAR‐body A was chosen for the further development of IgG1‐format Ars2 BAR‐bodies.

### 3.5 Expression of IgG1‐format Ars2 BAR‐bodies

Sequences of the heavy‐ and light‐chain BAR regions of clone 3 of the Fab‐format Ars2 BAR‐body A were cloned into a pSfi FLAG‐tag expression vector containing the constant regions of an IgG1 antibody. After transfection into HEK‐293 cells, IgG1‐format BAR‐bodies were detected using western blot analysis showing a molecular weight of approximately 150 kDa (Figure S3).

### 3.6 Binding properties of IgG1‐format Ars2 BAR‐bodies

Next, we assessed the ability of the IgG1‐format Ars2 BAR‐bodies to bind specifically to the B‐cell lymphoma cell lines U2932 and OCI‐Ly3 that are known to express Ars2‐reactive BCRs. HBL‐1, MAVER‐1, and Granta‐519 cells served as negative control. IgG1‐format LRPAP1 and neurabin‐I BAR‐bodies [15, 24]. were used as additional control. IgG1‐format LRPAP1 BAR‐bodies showed no binding to HBL‐1, U2932, OCI‐Ly3, or Granta‐519 cells but bound to MAVER‐1 cells, a cell line with LRPAP1‐reactive BCRs. IgG1‐format Ars2 BAR‐bodies, on the other hand, demonstrated specific binding to U2932 and OCI‐Ly3 cells but not to HBL‐1, MAVER‐1, or Granta‐519 cells. IgG‐format neurabin‐I BAR‐bodies did not bind to any investigated cell line (Figure 3).

### 3.7 Effector functions of IgG1‐format Ars2 BAR‐bodies

**FIGURE 4:**
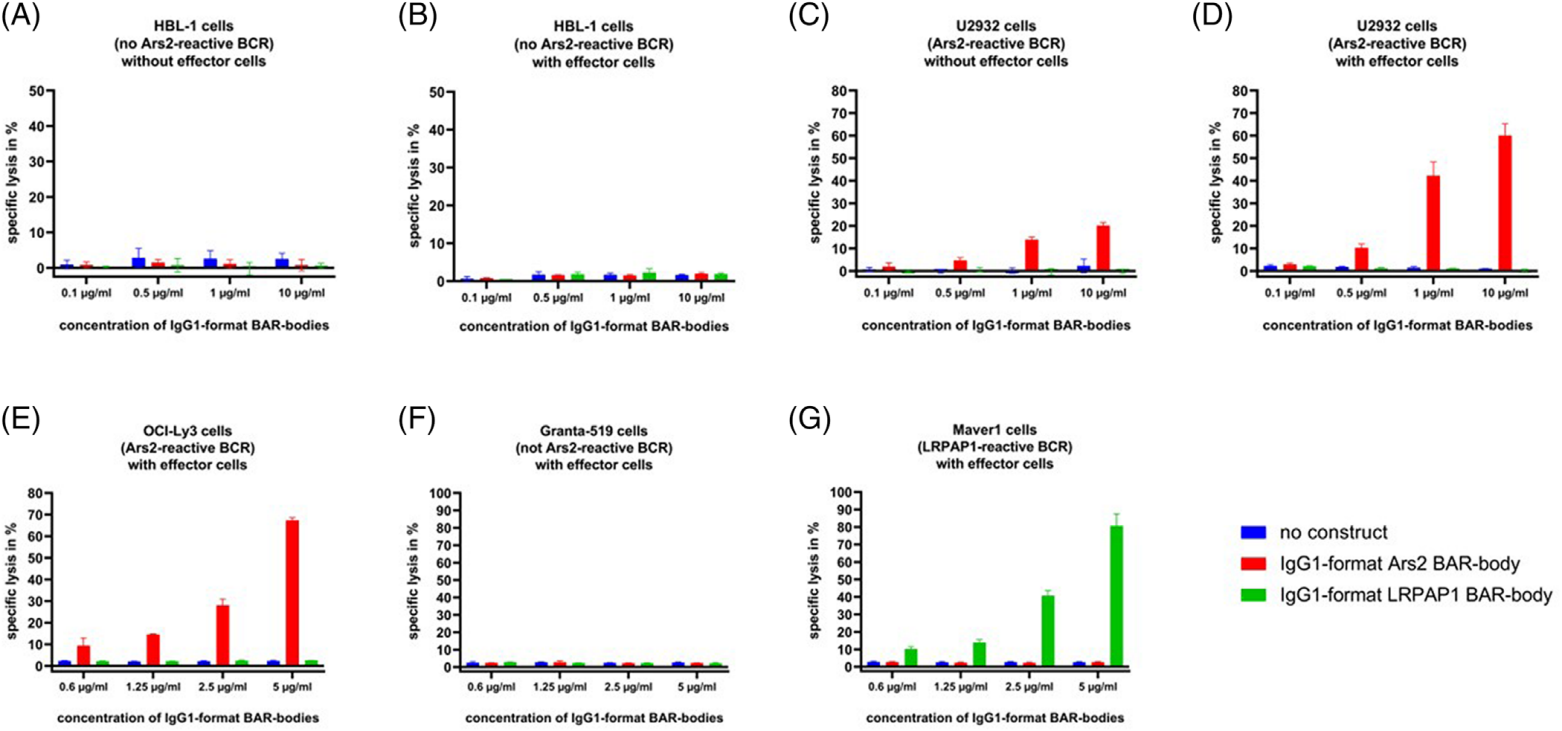
Cytotoxicity assays (LDH‐release) of IgG‐format BAR‐bodies incorporating Ars2 and LRPAP1 against diffuse large B‐cell lymphoma (DLBCL) cell lines U2932, OCI‐Ly3, and HBL‐1 as well as mantle cell lymphoma (MCL) cell lines Granta‐519 and MAVER‐1. LDH‐release assays with Ars2 BAR‐bodies on HBL‐1 and U2932 cells were performed with and without effector cells (peripheral blood mononuclear cells [PBMCs]). Both Ars2 and LRPAP1 incorporating IgG‐format BAR‐bodies had no effect on HBL‐1 cells without (A) or with (B) effector cells. Ars2, but not LRPAP1 BAR‐bodies, induced specific lysis in U2932 cells. Ars2 BAR‐bodies showed cytotoxic effects (specific lysis of approximately 20% at 10 μg/ml) on U2932 cells even without effector cells (C). This effect increases to a specific lysis of approximately 60% after the addition of PBMCs (D). Similar results could be obtained with OCI‐Ly3 cells as targets, which also express B‐cell receptors (BCRs) with Ars2‐reactivity (E). LRPAP1 BAR‐bodies had no effect on either U2932 or OCI‐Ly3 cells. The MCL cell line Granta‐519 was used as additional negative control (F). LRPAP1 BAR‐bodies and the LRPAP1‐reactive MCL cell line MAVER‐1 were used as positive control (G). All experiments were performed in triplicate (error bars).

IgG1‐format Ars2 BAR‐bodies were evaluated for their therapeutic potential using LDH‐release and annexin staining assays. U2932 and OCI‐Ly3 cells expressing Ars2‐reactive BCRs and HBL‐1, Granta‐519, and MAVER‐1 cells (all expressing BCRs that are not Ars2‐reactive) were used as target cells, and PBMCs were used as effector cells at an E:T ratio of 10:1. IgG1‐format LRPAP1 BAR‐bodies served as negative control. IgG1‐format Ars2 BAR‐bodies had no effect on HBL‐1 cells when incubated with or without effector cells at concentrations ranging from 0.1 to 10 μg/ml (Figure 4A,B). The same results were observed for IgG1‐format LRPAP1 BAR‐bodies. Incubation of IgG1‐format LRPAP1 BAR‐bodies with U2932 cells and either PBMCs or no PBMCs did not result in any relevant lysis (Figure 4C,D). IgG1‐format Ars2 BAR‐bodies induced specific lysis of up to 20% in U2932 cells even in the absence of effector cells (Figure 4C). This effect was dose‐dependent. In the presence of PBMCs as effector cells, dose‐dependent and specific lysis of U2932 cells by IgG1‐format Ars2 BAR‐bodies was increased to 60% at 10 μg/ml (Figure 4D). IgG1‐format Ars2 BAR‐bodies also induced dose‐dependent and specific lysis of OCI‐Ly3 cells of approximately 70% at 5 μg/ml (Figure 4E). The MCL cell lines Granta‐519 (BCR of unknown reactivity) and MAVER‐1 (BCR with LRPAP1 reactivity) served as negative and positive controls, respectively. IgG1‐format Ars2 BAR‐bodies had no effect on either cell line, whereas LRPAP1 BAR‐bodies induced specific lysis of MAVER‐1 cells but not in Granta‐519 cells in the presence of PBMCs (Figure 4F,G).

IgG format LRPAP1, Ars2, and neurabin‐I BAR‐bodies were tested for their potential to induce apoptosis in the absence of effector cells. In MAVER‐1 cells, the addition of LRPAP1 but not Ars2 or neurabin‐I integrating BAR‐bodies induced apoptosis in around 28% of cells with only 2% necrotic cells. Similarly, in OCI‐Ly3 and U2932 cells, the addition of the IgG‐format Ars2 BAR‐body without effector cells induced 10% and 17% apoptosis as compared to 2% and 1% necrosis, respectively (Figure S5). LRPAP1 or neurabin‐I integrating BAR‐bodies had no effect on OCI‐Ly3 and U2932 cells. No apoptosis or necrosis was observed in TMD8 and HBL‐1 cells after the addition of any IgG‐format BAR‐body (Figure S4).

## 4 DISCUSSION

Approximately 30% of patients with DLBCL relapse or have refractory disease after first‐line therapy [2]. In the recently published ZUMA‐7 trial, autologous stem cell transplantation was challenged by CAR T‐cell therapy with axicabtagene ciloleucel (axi‐cel) as a standard of care in refractory or early relapsed DLBCLs [25]. Further treatment options beyond first‐line exist, but many patients relapse even after CAR T‐cell therapy, and new therapeutic options are still needed [26, 27]

Here, we report on two new therapeutic formats that integrate the BCR antigen Ars2 to generate BAR‐bodies. In both, the bispecific and IgG1‐format BAR‐bodies, the BCR‐binding epitope of Ars2 replaces the antigen‐binding variable regions of a regular antibody to form the BAR region representing their targeting moiety to direct them to lymphoma cell BCRs.

The anti‐CD3/Ars2 construct led to higher maximum lysis at lower concentrations compared to anti‐CD16/Ars2. We believe that a combination of factors may be responsible for these results, such as differences in cytotoxic potential between NK‐ and T‐cells as well as differences in binding affinity of the incorporated single‐chain fragments. Notably, anti‐CD3/Ars2 constructs start to confer cytotoxic effects at concentrations of 0.1 μg/ml. For blinatumomab, a commercially available CD19/CD3 bispecific antibody, much lower concentrations of down to 0.1 ng/ml have been reported as effective doses [28]. We cannot definitively explain these differences but believe that factors like production purity of our bispecific constructs, BCR density on the cell surface, and affinity of a given BAR to its respective BCR may contribute to this observation.

Bispecific Ars2 BAR‐bodies have a molecular weight of about 55 kD. Construct size plays an important role in pharmacokinetics of drugs. For blinatumomab, which is of similar size, an elimination serum half‐life time of 1.25 h is described [29, 30]. This relatively short period of time has the advantage of exact dosing that is important in the management of adverse events. On the other hand, the rapid elimination of blinatumomab requires its continuous intravenous infusion.

In contrast, due to their molecular weight of approximately 150 kDa, the pharmacokinetics of full‐length IgG1 antibodies like rituximab are completely different and result in elimination serum half‐life times of 25–30 days [31, 32]

The optimal therapeutic format to integrate Ars2 into has not been determined and we aimed to generate additional Ars2‐containing constructs resembling IgG1‐format antibodies. IgG1‐format Ars2 BAR‐bodies induced relevant specific lysis in OCI‐Ly3 and U2932 DLBCL cells when incubated with PBMCs. Ars2 BAR‐bodies in IgG1 format had no effect on HBL‐1 lymphoma cells, which are also DLBCL cells but expressing a BCR of unknown reactivity and also not on both MCL cell lines MAVER‐1 and Granta‐519. IgG‐format LRPAP1 BAR‐bodies induced cytotoxic effects in MAVER‐1 cells expressing LRPAP1‐reactive BCRs, but they had no effect on any of the other cell lines. These highly selective cytotoxic effects show that BAR‐bodies target lymphoma cells via their BCR with highest specificity.

Interestingly, IgG1‐format Ars2 BAR‐bodies led to specific and dose‐dependent lysis in Ars2‐reactive U2932 lymphoma cells even in the absence of effector cells. Cytotoxic effects of Ars2 IgG1‐format BAR‐bodies without effector cells did not reach the level reached in the presence of PBMCs as effector cells but were still reproducible with 20% lysis at 10 μg/ml. It can be speculated that this direct cytotoxic effect of IgG1‐format Ars2 BAR‐bodies is conveyed by the mechanism of activation‐induced cell death in B‐lymphocytes (AICD) after cross‐linking of two adjacent BCRs by IgG1‐format Ars2 BAR‐bodies that present four BCR‐binding BAR regions [33]. AICD is thought to occur when the BCR pathway of B lymphocytes is activated in the absence of additional survival signals like CD40 or IL‐4R engagement. These initial findings could be confirmed as annexin staining of lymphoma cells after the addition of IgG‐format BAR‐bodies led to highly specific apoptosis rather than the necrosis of lymphoma cells with matching BCRs even without the addition of effector cells (Figures S4 and S5).

Ars2‐integrating BAR‐bodies may represent an additional therapeutic option for patients with DLBCL. Because Ars2‐reactive BCRs are expressed mostly in ABC‐type DLBCLs and account for roughly 25% in this subgroup, Ars2‐containing BAR‐bodies would be applicable only in a fraction of DLBCL patients. We believe that this is an inherent problem of personalized medicine: treatment approaches that are highly specific to a certain antigen or pathway with little toxicity can very likely not be used in the majority of patients. Still, the BAR‐body concept may be a powerful tool as an additional future treatment option for B‐cell lymphomas considering that several B‐cell lymphoma autoantigens have already been identified, including LRPAP1, neurabin‐I/SAMD14, and Ars2. The identification of additional DLBCL BCR autoantigens would allow for a wider use of BAR‐bodies in a larger percentage of patients.

## AUTHOR CONTRIBUTIONS

Maximilian Kiefer, Moritz Bewarder, Lorenz Thurner, and Konstantinos Christofyllakis wrote the manuscript. Moritz Bewarder, Lorenz Thurner, Klaus‐Dieter Preuss, Maximilian Kiefer, and Michael Pfreundschuh designed and supervised the experiments. Maximilian Kiefer, Evi Regitz, and Natalie Fadle performed the experiments. Moritz Bewarder, Maximilian Kiefer, and Konstantinos Christofyllakis are responsible for data analysis. Dominic Kaddu‐Mulindwa, Joerg Thomas Bittenbring, Frank Neumann, Igor Kos, Onur Cetin, Vadim Lesan, and Markus Hoth revised the manuscript.

## CONFLICTS OF INTEREST

The authors declare that the research was conducted in the absence of any commercial or financial relationships that could be construed as a potential conflict of interest.

## ETHICS STATEMENT

The study had been approved by the local ethics committee (Aerztekammer des Saarlandes 12/13).

## Supporting information

Supporting Information

## Data Availability

The data that support the findings of this study are available from the corresponding author upon reasonable request.
