# Supplementary material for: Ars2‐containing bispecific, Fab‐ and IgG1‐format BAR‐bodies to target DLBCL cells
Source: EJHaem. 2022 Dec 27;4(1):125–34. doi: 10.1002/jha2.635 (PMC9928785; doi:10.1002/jha2.635)
Supplement: Supplementary file 1 — Supporting Information [file JHA2-4-125-s001.docx]

**Supplemental Figures**


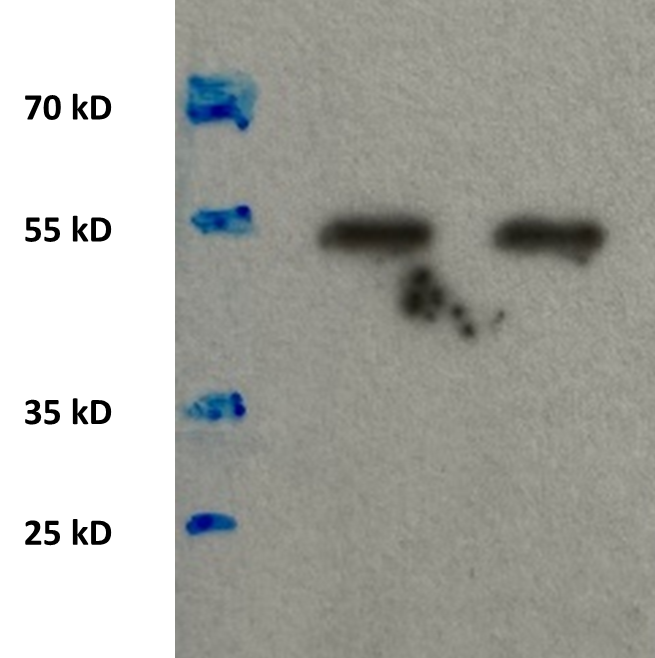


Supplemental Figure 1: bispecific Ars2 BAR-bodies anti-CD3/Ars2 (lane 1) and anti-CD16/Ars2 (lane 2).


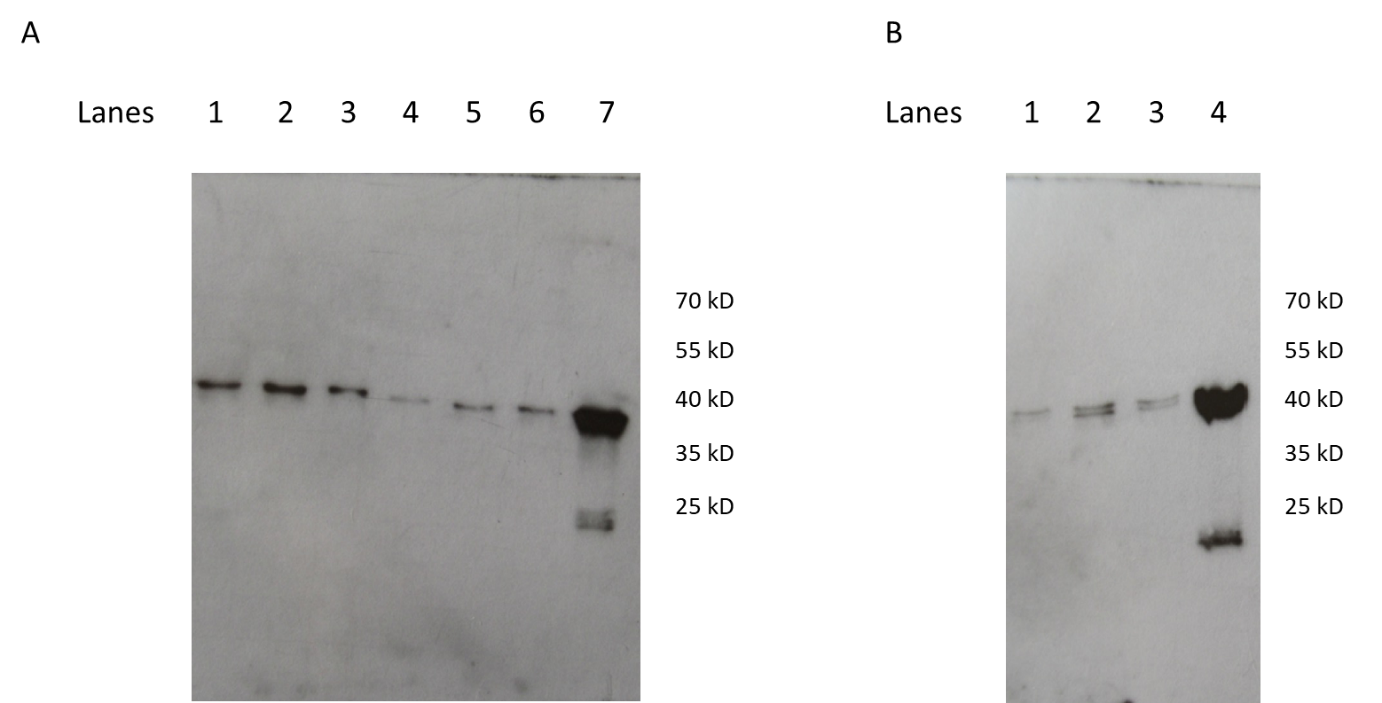


Supplemental Figure 2: Western blot analysis of Fab-format Ars2 BAR-bodies. (A) Fab-format Ars2 BAR-bodies A (BAR region: aa 343-466) and B (BAR region: aa 290-410). Lanes 1, 2 and 3 show the Fab-format Ars2 BAR-body A (three different clones), lanes 4, 5, 6 show three different clones of the Fab-format Ars2 BAR-body B. Lane 7 shows the bispecific anti-CD3/Ars2 BAR-body as control. All constructs are running with the 40 kDa band of the marker as expected. (B) Fab-format Ars2 BAR-body C (BAR region: aa 260-375). Lanes 1, 2 and 3 show three clones of Fab-format Ars2 BAR-body C with a molecular size of 40 kDa and lane 4 shows the bispecific anti-CD3/Ars2 BAR-body as control.


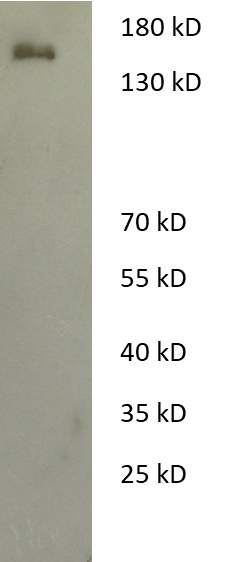


Supplemental Figure 3: Western blot analysis of the IgG1-format Ars2 BAR-body: The IgG1-format Ars2 BAR-body is running between 180 kDa and 130 kDa suggesting a molecular weight of about 150 kDa.


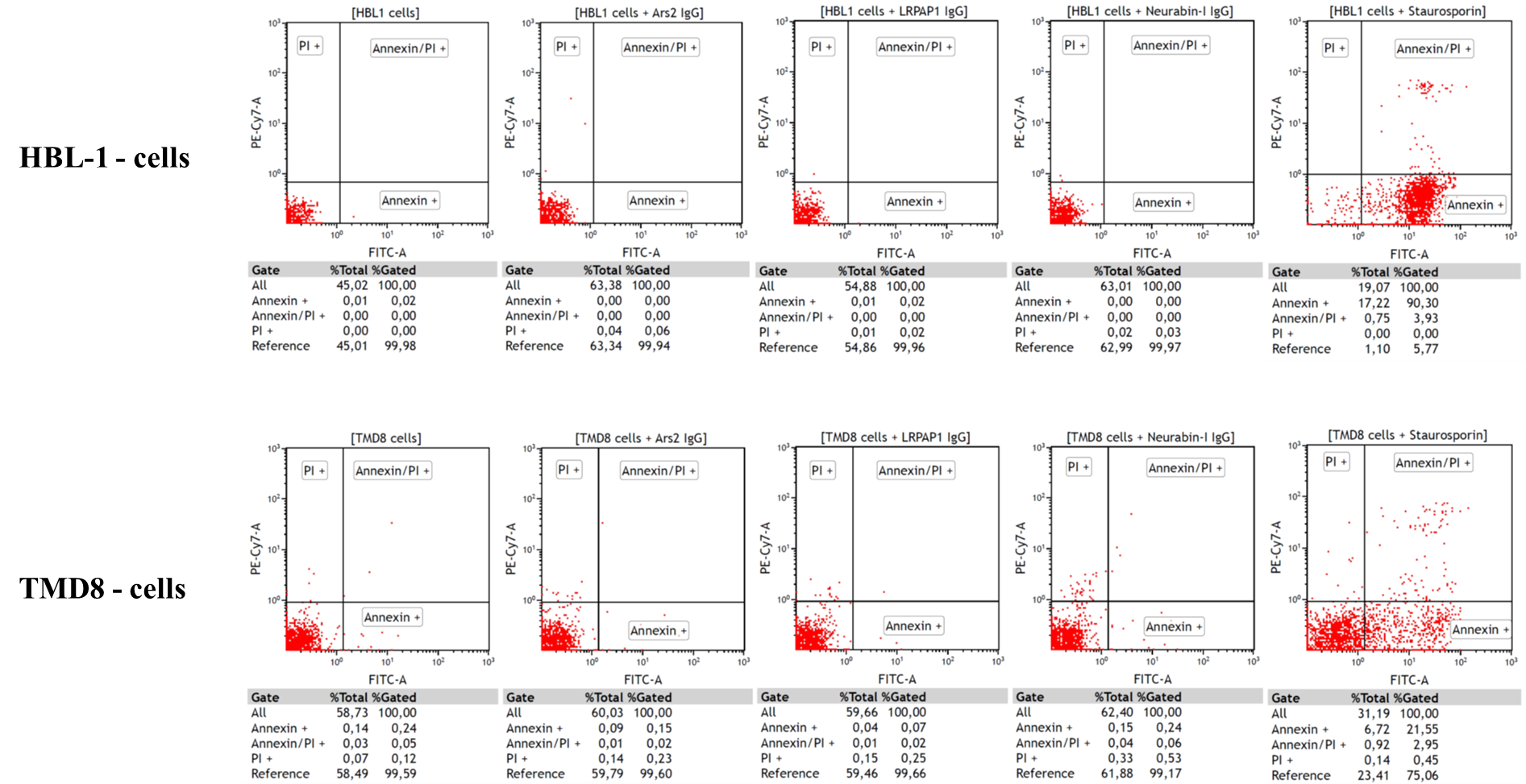


Supplemental Figure 4: IgG-format BAR bodies incorporating Ars2, LRPAP1 and Neurabin-I were tested via flow cytometry for their potential to induce either apoptosis (shift on the x-axis, annexin staining) or necrosis (shift on the y-axis, propidium iodide staining) on HBL-1 and TMD8 cells without the addition of effector cells. Staurosporine was used as positive control for the detection of apoptosis. No BAR-body had any effect on both cell lines which was to be expected as they express BCRs of unknown reactivity. After the addition of staurosporine appr. 90 % of HBL-1 cells and 22 % of TMD8 cells stain positive for annexin, i.e. undergo apoptosis.


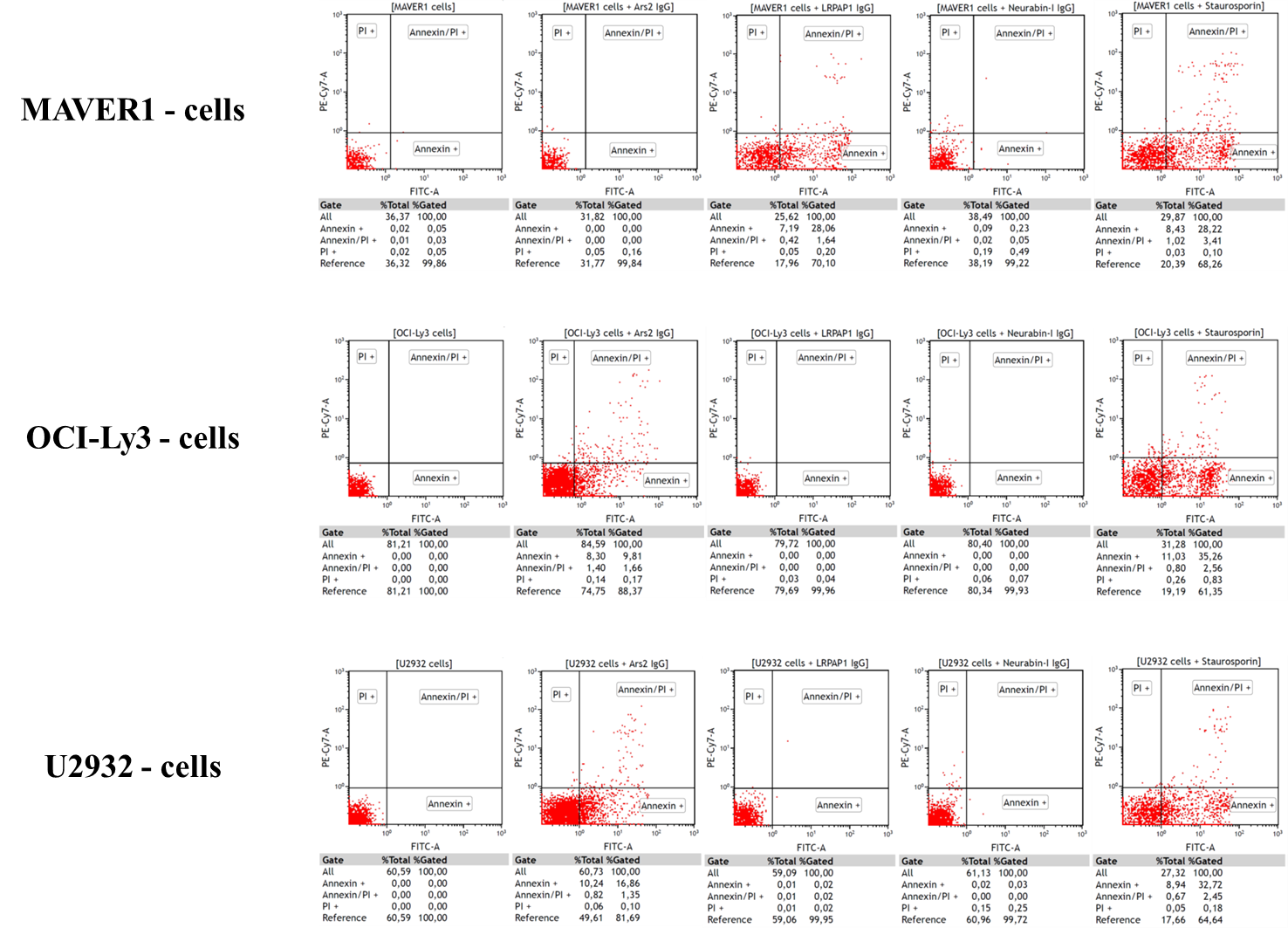


Supplemental Figure 5: IgG-format BAR bodies incorporating Ars2, LRPAP1 and Neurabin-I were tested via flow cytometry for their potential to induce either apoptosis (shift on the x-axis, annexin staining) or necrosis (shift on the y-axis, propidium iodide staining) on MAVER-1, OCI-Ly3 and U2932 cells. Staurosporine was used as positive control for the detection of apoptosis. After the addition of staurosporine appr. 28 % of MAVER-1 cells, 35 % of OCI-Ly3 cells and 33 % of U2932 cells stain positive for annexin, i.e. undergo apoptosis with around 3 % necrotic cells. In MAVER-1 cells, the addition of the IgG-format LRPAP1 BAR-body without effector cells induces apoptosis in around 28 % of cells with only 2 % necrotic cells. Ars2 or neurabin-I integrating BAR-bodies had no effect on MAVER-1 cells. In OCI-Ly3 cells, the addition of the IgG-format Ars2 BAR-body without effector cells induces apoptosis in around 10 % of cells with 2 % necrotic cells. LRPAP1 or neurabin-I integrating BAR-bodies had no effect on OCI-Ly3 cells. In U2932 cells, the addition of the IgG-format Ars2 BAR-body without effector cells induces apoptosis in around 17 % of cells with 1 % necrotic cells. LRPAP1 or neurabin-I integrating BAR-bodies had no effect on U2932 cells.

**Supplemental Methods**

*Cloning concept of bispecific Ars2 BAR-bodies*

Previous experiments determined the amino acids (aa) 343 to 375 of Ars2 as the binding epitope for Ars2-reactive B-cell receptors.^1^ Variable light-chain (VL) and variable heavy-chain (VH) domains of the anti-CD3 OKT 3 hybridoma^2^ and of the anti-CD16 3G8 hybridoma^3^ cell lines were cloned into a pcDNA 3.1 vector (Invitrogen, Carlsbad, California, USA) by standard cloning techniques, followed by the DNA sequence of the ARS2 BCR-binding epitope. The single-chain fragment (scFv) containing the variable heavy and light chains of the anti-CD16 hybridoma, linked by a glycine-serine linker, was obtained from Genscript (GenScript USA Inc., 860 Centennial Ave. Piscataway, NJ 08854, USA). Primers used for PCR amplification of the anti-CD3 scFv and the ARS2 epitope are shown in *Table 1*. VH and VL were linked by a glycine-serine linker, as was the ARS2 epitope to VL, resulting in a VH-(GlySer)4-VL-(GlySer)3-ARS2 peptide chain. The ARS2 epitope was tagged with a polyhistidine tail for subsequent detection and purification.

*Cloning concept of Fab-format Ars2 BAR-bodies*

In order to replace the variable regions of Fab antibodies with a protein structure that contains the BCR-binding epitope of Ars2, three different Ars2 sequences were amplified by PCR. Each sequence had a length of approximately 120 aa and contained the BCR-binding epitope of Ars2 (aa 343-375). These sequences were cloned separately into a pCR 2.1 vector (ThermoFisher Scientific, Karlsruhe, Germany). Sequences were inserted at the position of the variable heavy chain (VH) and variable light chain (VL) regions of the pCES-1 vector. The former variable antibody regions are now called BAR-regions. Three versions of Fab-format BAR-bodies were generated with the Ars2 epitope at different positions in the BAR-region: version A (aa 343-466), version B (aa 290-410), version C (aa 260-375). Version A contains the Ars2-binding epitope located at the 5’ end of ARS2. Primers and restriction sites are content of *Table 2*.

*Cloning strategy for the IgG1-format Ars2 BAR-body*

A pCR 2.1 vector (ThermoFisher Scientific, Karlsruhe, Germany) was modified by integrating the variable and the constant domains of an immunoglobulin heavy chain (VH and CH1-CH3), a furin cleavage site, a 2A peptide sequence, a light chain variable domain (VL) and a light chain constant domain (CL) from an IgG1 immunoglobulin.^4^ The Ars2 epitope-containing BAR-region of the Fab-format Ars2 BAR-body (version A) was cloned into the pCR 2.1 vector in front of CH1-CH3 to replace the VH domain and additionally in front of CL to replace the VL domain. The modified heavy chain was cloned into a pSfi FLAG-tag expression vector. This vector was derived from the pEGFP-C1 vector by replacing the eGFP ORF with a FLAG-tag (Clontech, Mountain View, California, USA). The modified light chain was cloned into the pSfi FLAG-tag expression vector. Primers and restriction sites are shown in *Table 3*.

*Expression, purification and detection of bispecific Ars2 BAR-bodies*

Diluted plasmid DNA (1 µg, pcDNA 3.1 vector) was incubated with 3 µl X-tremeGENE HP DNA Transfection Reagent (Sigma-Aldrich, Missouri, USA). After 10 minutes of incubation the mixture was added to HEK-293 cells in RPMI 1640 medium (Pan-Biotech GmbH, Aidenbach, Germany). The cell suspension was then incubated for 48 hours at 37°C and 5% CO2. The histidine-tag of the bispecific constructs was used for isolation and purification by cobalt-based „Immobilized Metal Affinity Chromatography“ (IMAC). In short, transfected cells were harvested and lysed in 10mM TRIS pH8 buffer for 30 minutes at 4°C. Following this, 50 µl of TALON beads (ThermoFisher Scientific; 168 Third Avenue, Waltham, MA USA 02451) were added and incubated for 30 minutes on ice. After 3 washing steps, proteins were eluted with 150 mM imidazole and re-buffered in PBS. Isolated protein was loaded onto a 10% SDS-PAGE gel for separation. It was then blotted onto a PVDF membrane using a transblot semi-dry transfer cell (Bio-Rad Laboratories GmbH, Hercules, California, USA). After blocking in 10% non-fat dry milk and PBS at 4°C, the membrane was incubated with 1:2000 murine anti-His antibody (Qiagen, Hilden, Germany). After 3 cycles of washing with 0.1% Triton X-100 in TBS, the membrane was incubated with 1:3000 HRP-labeled anti-mouse IgG antibody (Bio-Rad Laboratories GmbH, Hercules, California, USA). For final detection on hyperfilm ECL (Merck, Darmstadt, Germany), the PVDF membrane was incubated with chemiluminescence reagent LumiGLO^®^ by Cell signaling Technology (Massachusetts, USA).

*Expression, purification and detection of the Fab-format Ars2 BAR-body*

Fab-format Ars2 BAR-body plasmids were transformed into TG1 *e. coli* bacteria. For expression, TG1 bacteria were cultured in 50 ml TY medium to a cell density of 0.6 - 0.8 measured at 600nm. Next, 1 mM isopropylthiogalactosid (IPTG) was added, followed by incubation for 4 hours at 30°C. Finally, after centrifugation at 14,300 rpm for 1 minute, the Fab-format Ars2 BAR-bodies were purified using an integrated histidine-tag (his-tag) and cobalt-based IMAC (TALON beads (ThermoFisher Scientific; 168 Third Avenue, Waltham, MA, USA). After 3 cycles of washing, the his-tag conjugated protein was eluted with 200µl 150mM imidazole. Detection was performed by western blot analysis as described above.

*Expression, purification and detection of the IgG1-format Ars2 BAR-body*

The IgG1-format Ars2 BAR-body containing pSfi FLAG-tag vector was transfected into HEK-293 cells. Therefore, 1 µg plasmid DNA was incubated with 3 µl X-tremeGENE HP DNA Transfection Reagent (Sigma-Aldrich, Missouri, USA) for 10 minutes. This mixture was added to HEK-293 cells cultured in RPMI 1640 medium (Pan-Biotech GmbH, Aidenbach, Germany) and incubated for 48 hours at 37°C and 5% CO2. For purification, 50 ml of supernatant was incubated with 150 µl ANTI-FLAG® M2 Affinity Gel (Sigma-Aldrich, St. Louis, USA) at 4°C overnight. After centrifugation at 3,500 rpm for 10 minutes and 3 cycles of washing with PBS pH7 the protein was eluted with 200 µl 0,2M glycine. After centrifugation at 12,000 rpm, the supernatant contains the purified protein. For pH neutralization 20 µl 1M natrium-hydrogen-phosphate was added. Glycine was removed using a 10 kDa dialysis membrane (SERVA Electrophoresis GmbH, Heidelberg, Germany) against PBS pH7 for 12 hours at 4°C. Western blot analysis was used for detection of IgG1-format Ars2 BAR-bodies (ANTI-FLAG® M2 antibody (Merck, Karlsruhe, Germany), Goat Anti-Mouse IgG (H+L) - HRP Conjugate).

*Apoptosis assay: Staining of Lymphoma cell lines with annexin and propidium iodide*

For the analysis of apoptosis and to differentiate apoptosis from necrosis, 1 × 106 cells/well of OCI-LY3, U2932, HBL-1, TMD-8 and Maver1 cells were treated with neurabin-I, LRPAP1 and Ars2 BAR-bodies in the IgG-format (0.5 µg/ml) or staurosporine (0.2 µM) for 24 hours at 37°C, 5% CO2. Cells were washed twice with phosphate-buffered saline and resuspended in 500 µL binding buffer. 5 µL AnnexinV-FITC and 10 µL propidium iodide were added to each cell suspension and incubated for 10 minutes at room temperature, followed by analysis by flow cytometry.

References

1. Thurner L, Hartmann S, Bewarder M, Fadle N, Regitz E, Schormann C, et al. Identification of the atypically modified autoantigen Ars2 as the target of B-cell receptors from activated B cell–type diffuse large B-cell lymphoma. Haematologica [Internet]. 2020 Jul 16 [cited 2021 Feb 22];haematol.2019.241653. Available from: https://pubmed.ncbi.nlm.nih.gov/32675228/

2. Kung PC, Goldstein G, Reinherz EL, Schlossman SF. Monoclonal Antibodies Defining Distinctive Human T Cell Surface Antigens. Science (80- ) [Internet]. 1979 Oct 19 [cited 2022 Apr 17];206(4416):347–9. Available from: https://www.science.org/doi/abs/10.1126/science.314668

3. Fleit HB, Wright SD, Unkeless JC. Human neutrophil Fc gamma receptor distribution and structure. Proc Natl Acad Sci U S A [Internet]. 1982 [cited 2022 Apr 17];79(10):3275. Available from: /pmc/articles/PMC346398/?report=abstract

4. Freitag J, Heink S, Roth E, Wittmann J, Jäck HM, Kamradt T. Towards the generation of B-cell receptor retrogenic mice. PLoS One. 2014 Oct 8;9(10).
